# Viruses and Breast Cancer

**DOI:** 10.3390/cancers2020752

**Published:** 2010-04-30

**Authors:** James S. Lawson, Benjamin Heng

**Affiliations:** School of Biotechnology and Biomolecular Sciences, University of New South Wales, Sydney, Australia; E-Mail: z3174934@unsw.edu.au (B.H.)

**Keywords:** breast cancer, viruses, etiology, human papilloma virus, mouse mammary tumor virus, Epstein-Barr virus, bovine leukemia virus

## Abstract

Viruses are the accepted cause of many important cancers including cancers of the cervix and anogenital area, the liver, some lymphomas, head and neck cancers and indirectly human immunodeficiency virus associated cancers. For over 50 years, there have been serious attempts to identify viruses which may have a role in breast cancer. Despite these efforts, the establishment of conclusive evidence for such a role has been elusive. However, the development of extremely sophisticated new experimental techniques has allowed the recent development of evidence that human papilloma virus, Epstein-Barr virus, mouse mammary tumor virus and bovine leukemia virus may each have a role in the causation of human breast cancers. This is potentially good news as effective vaccines are already available to prevent infections from carcinogenic strains of human papilloma virus, which causes cancer of the uterine cervix.

**Note:** In this paper we have followed the convention that malignancies of the human breast are referred to as breast cancer or breast tumors and malignancies of mouse mammary glands are referred to as mammary tumors.

## 1. Introduction

The identification of the causes of breast cancer is a crucial research issue for the development of effective prevention and treatment strategies. The aim of this review is to demonstrate that oncogenic viruses may have causal roles in human breast cancer. The main candidate viruses are human papilloma virus (HPV), mouse mammary tumor virus (MMTV), Epstein-Barr virus (EBV) and bovine leukemia virus (BLV). Each of these viruses has known oncogenic potential and all have been identified in normal and malignant human breast tissues. The past priority has been to confirm the identity of these viruses in breast tumors. The new priority is to determine whether or not they are causal rather than innocuous passengers invading pre-existing malignant tissues.

The formal search for the causes of human breast cancer began over 100 years ago. Despite cancer research expenditures exceeding $100 billion US dollars during the past 30 years, that hunt, and the basic causes of breast cancer, remain elusive [1]. However, sound progress has been achieved. The risk factors, as distinct from causal factors for breast cancer, have been well identified [2,3]. In addition, patterns of genetic susceptibility, again as distinct from causal patterns, have been both specifically and broadly defined [4]. The only specific known cause of breast cancer is exposure to radiation such as Japanese victims of the atomic bomb explosions, among whom young females were at special risk [5].

## 2. Interesting Epidemiological Observations

There are several fundamental epidemiological features of breast cancer directly related to finding the underlying causes. These are: (i) although breast cancer occurs in men it is an overwhelmingly female disease; (ii) the incidence and mortality of breast cancer is four-to-five times greater among Western than Asian women, but these differences rapidly diminish following migration to high risk countries; (iii) MMTV positive house mice are more common in countries with high breast cancer incidence; (iv) EBV associated infectious mononucleosis is more common in Western than Asian countries and correlates with the high incidence of breast cancer. These epidemiological features are important because they indicate that external factors, as distinct from endogenous genetic and other factors, must have influential roles in the etiology of breast cancer. We shall consider these epidemiological features in more detail:

*(i) Breast cancer is an overwhelmingly female disease.* Many known increased risk factors are estrogen and progesterone related [6]. Estrogen and progesterone are fundamental to being female. Early age menarche, late age menopause and postmenopausal obesity increase the lifetime exposure to estrogens. Estrogen and progesterone hormone replacement therapy increases the risk of invasive breast cancer by over 25% in Western women [7]. It seems likely the reason for the increased risk is the increased exposure to these hormones. The increased exposure to these hormones suggests they act as continuous growth promoters following initiation of breast cancer by other factors. While the mechanisms are not known, the implication is that these sex hormones clearly have a role in breast cancer.

*(ii) Breast cancer as a migration risk.* The four-to-five-fold differences in breast cancer risk between Asian and Western women has been a consistent phenomena since reliable data became available over 50 years ago. The increased risk following migration has also been a consistent phenomenon (Table 1).

**Table 1:** Breast cancer incidence per 100,000 females (age adjusted) for US, Chinese and Japanese in home countries and after migration [8].

	Born US, China, Japan	Born China, Japan migrate to US	Born in US
US	159		159
Chinese	19	47	59
Japanese	19	41	75

The incidence of breast cancer parallels food consumption patterns (Table 2). High consumption of energy and fats between countries has consistently correlated with breast cancer incidence and mortality for over 50 years. This is despite the many studies which show there is no such relationship [9]. In our view, this may be because most studies have been of women from Western countries whose food consumption, among the lowest consumers, is well above that of highest food consumers in some Asian countries where food consumption does significantly correlate with breast cancer risk [10]. Serum sex hormone levels among Western women are double those of Asian women and these levels are directly related to food consumption patterns [11]. It is also known that food consumption patterns alter following migration as Asian festival food, which includes chicken, pork and beef, gradually becomes part of regular food consumption patterns [12]. The implication is that food-dependent sex hormone levels and increased adiposity (adipose tissues generate estrogens) appear to be associated with increased risk of breast cancer.

**Table 2:** Breast cancer mortality and food consumption: International comparisons. Breast cancer mortality rates for the year 2002 per 100,000 females-age adjusted [13,14].

	Breast cancer mortality	Calories/day/capita	Total fats/capita
USA	19.0	3,774	157
UK	24.3	3,412	139
Australia	18.4	3,054	131
Japan	8.3	2,761	85
South Korea	4.4	3,058	77
China	5.5	2,951	90

In addition to changes in food consumption patterns following Asian migration to the US and other Western countries, social and behavioral changes relevant to breast cancer also commonly occur. These changes include sexual behavior that may lead to increased risk of HPV infections, later age full term pregnancy which increases risk of breast cancer and the use of hormone replacement therapy and increased alcohol consumption. However, it is our view that the most influential change following such migration is food consumption patterns. We base this opinion on the greatly increased risk of prostate cancer following Asian migration to the West, such risk is obviously not influenced by changes in sexual behavior and fertility patterns. We should add that our opinion is not generally shared by the scientific community.

An intriguing phenomenon associated with risk of breast cancer and migration, is the association of later age infections with EBV and associated clinical symptoms of infectious mononucleosis (glandular fever). Despite the almost universal infection by EBV throughout the world, symptomless EBV infections predominantly occur during infancy in developing and population dense countries, in contrast to EBV infections in Western countries, which also occur in infancy and childhood, but more commonly with clinical symptoms in teenage and young adulthood [15]. Therefore, infections with EBV are similar to viral hepatitis and poliomyelitis, both of which cause symptoms if the infection is delayed past early childhood. Delayed EBV infection is recognized as a causal factor in some but not all Hodgkin’s lymphomas and there is suggestive evidence that delayed EBV infection may be associated with breast cancer [15]. This observation is in accord with the lesser increase in breast cancer incidence among migrants born in Asia but who migrate to the US, as compared to the much larger increase in breast cancer incidence among Asians born in the US [15]. These data are shown in Table 1.

(iii) *Breast cancer and the house mouse.* The highest incidence of human breast cancer worldwide occurs in lands where *Mus domesticus* is the resident native or introduced species of house mouse [16]. *Mus domesticus* has the highest known concentration of MMTV of all mouse species. In many countries including the US, mouse excreta are allowed in wheat and other cereals and may be a means of transmission as part of the human food chain. The implication is that MMTV crosses species and may cause some human breast cancers.

(iv) *Breast cancer heterogeneity offers a causal clue.* The cell structure of breast lobules is simple. Surrounding the breast lobule lumen there is a luminal (internal) layer of epithelial cells, which can produce milk, and a basal (external) layer of myoepithelial cells, which may contract and express milk under hormonal influences such as oxytocin. Despite this simplicity, there are a wide range of human breast cancer types classified by histological characteristics (over 30 histological types according to the World Health Organisation classification). These are listed in a very simple classification in Table 3. The underlying molecular characteristics do not exactly match this simple classification, but there are broad parallels. The molecular patterns characteristic of invasive ductal carcinoma (IDC) are different to those of the various invasive lobular carcinomas (ILC) and *in situ* carcinomas have a mixture of ductal and lobular carcinomas [17]. In turn the histological characteristics of IDC have some similarities to HPV positive breast tumors and ILC has similarities to MMTV-like sequence positive human breast tumors [18,19]. While these virus and histological characteristic parallels are speculative, when considered in the context of the virus and breast cancer related evidence below, they have potential biological relevance.

**Table 3:** Breast cancer–histological and molecular types.

	Proportion of breast cancers	10 year survival	Putative viral type [18,19]
Invasive ductal carcinoma (IDC)	60%	50%	HPV/EBV
Invasive lobular carcinoma (ILC)	5%	50%	MMTV
Invasive lobular carcinoma–special types (medullary, tubular, neuroendocrine)	10%	75–90%	MMTV
Non- invasive (ductal carcinoma *in situ -* DCIS)	25%	90%	MMTV/HPV/EBV

The known risk factors are seemingly bizarre but most are associated with exposure to estrogens and other hormones [6]. The most important risk factors include being an older aged Western female (*i.e*., risk factors of age, gender and Western culture and lifestyle), so much so, that up to one in eight Western women will develop breast cancer during their lifetime. Other risk factors include early age menarche and late age menopause, above average birth weight, weight gain and obesity in the immediate postmenopausal years and the use of hormone replacement therapy. Early age full term pregnancy offers some protection against breast cancer, but the underlying mechanism remains obscure. Of great interest is the four-to-five-fold greater risk of breast cancer among Western as compared to Asian women, a difference which lessens following migration of Asian women to the US and other Western countries [8]. See Table 1. 

In a prospective study of 74,647 Californian school teachers aged 50 years or older, Marshall *et al.* conclusively demonstrated that post menopausal use of estrogens and progesterones is associated with a 26% increase of invasive (26% associated with estrogen alone and 45% associated with estrogen and progesterone hormone replacement therapy), but not *in situ*, breast cancer [7]. Their observations do not imply that estrogens are carcinogenic but they do have a role by increasing risk of breast cancer. The underlying mechanisms for this role are not known but we have shown that estrogen receptor expression is greatly increased in obese postmenopausal women who use hormone replacement therapy and we have also shown that estrogen receptor expression in normal breast tissues of Western women is much higher than in low risk for breast cancer Japanese women [20,21]. Abnormally high estrogen receptor expression is a feature of many breast cancers whose progression is retarded by inhibitors of estrogen such as tamoxifen.

Because of the increased risk of breast cancer associated with use of hormone replacement, it is not possible to quantify the progress that has been made in reducing breast cancer mortality. The reduction in breast cancer mortality is mainly due to three factors: the widespread use of tamoxifen (which inhibits the influences of estrogen), the development and use of Herceptin (which is a monoclonal antibody that interferes with the HER2/neu receptor, thus selectively inhibiting the growth of breast cancer cells in about 25% of breast tumors) and mammography screening programs which has allowed the early diagnosis and treatment of many breast cancers.

There are three proposed mechanisms of how estrogens influence breast carcinogenesis: receptor-mediated hormonal activity, cytochrome P450 (CYP)-mediated metabolic activation, and induction of aneuploidy (chromosome abnormality) by estrogen itself [22,23,24,25,26]. Receptor mediated hormonal activity, in general, is related to stimulation of cellular proliferation. This creates opportunities for accumulation of genetic damages leading to carcinogenesis. Cytochrome P450 (CYP)-mediated metabolic activation involves pathways that generate free radicals and intermediate metabolites that can cause oxidative stress and genomic damage directly. Additional evidence is required to determine whether estrogens are carcinogenic for human breast cancer.

Postmenopausal weight gain has been shown to increase the risk of breast cancer. This appears to be due to higher levels of circulating estrogen, synthesized by adipose tissues, as well as lower sex hormone binding globulin levels, which allows more bioavailable estrogen [26].

Micro-array technology has been widely used during the past decade to identify a wide spectrum of genes associated with breast cancer [4]. These studies have shown that there is a reasonably consistent gene expression pattern associated with some uncommon, mainly invasive lobular carcinomas, but these advances have not so far been clinically useful [17]. Some women have a genetic predisposition to develop breast cancer, particularly those who carry mutated breast cancer genes *BRCA-1* and *2*.

## 3. Viruses and Breast Cancer

The possibility that viruses may have a role in breast cancer etiology was initiated in 1936 by John Bittner and colleagues at the Jackson laboratories in Maine [27]. They observed that mouse milk contained an unknown factor, which caused mammary tumors in their pups when they grew to adulthood. This unknown factor was later identified as mouse mammary tumor virus.

In the early years of the War on Cancer, introduced in 1971 by President Richard Nixon of the US, there was great interest in the idea that viruses may cause breast cancer. Little progress was made, however, to prove or disprove any links between viruses and breast cancer, and both research interest and supporting finance in this field had almost ceased by 1990. However, dramatic progress was made in the development of biological research techniques. Wonderful basic discoveries were made such as the role of oncogenes in the development of cancers (oncogenes are normal growth related genes that induce cancer when expressed out of control) and *p53* in tumor suppression and apoptosis (*p53* is a gene which normally helps maintain the normal growth control or death of cells but which also acts as an oncogene when mutated). The most influential developments were the conception of polymerase chain reaction (PCR) by Kary Mullis in 1983, the development of monoclonal antibodies by Georges Kohler, Cesar Milstein and Niels Jerne in 1975 and automated gene sequencing by Frederick Sanger and coworkers in the United Kingdom and Walter Gilbert and Allan Maxam in the US. There has been much controversy about who deserves the credit for these technical developments but, despite such conflicts and court cases over patent rights, the Nobel selection Committee has awarded prizes to all of the above scientists.

The availability of these new techniques enabled Di Lonardo *et al.* in 1992 to identify high risk HPV in breast cancers among Italian women and Horiuchi *et al.* in 1994 to identify EBV in breast cancers among Japanese women [28,29]. In 1995, Beatriz Pogo and colleagues identified MMTV in human breast cancers among New York women [30]. The identification of MMTV in humans was difficult because MMTV DNA sequences are similar to human endogenous retrovirus sequences (HERVs) and can also express cross reacting proteins [31]. The timing of these developments is no accident as they were made possible by the new technologies. More recently, using the new techniques, Gertrude Buehring has identified evidence of bovine leukemia virus (BLV) related DNA in breast cancers among women in the US [32,33,34].

These initial identifications of viruses with known oncogenic capacities in human breast cancers have been confirmed in many countries and in different laboratories [35]. Our group has recently confirmed the unambiguous presence of high risk HPV and MMTV-like sequences in invasive and *in situ* breast tumors [19,36]. In addition, we have demonstrated the presence of HPV associated pre-malignant koilocytes in normal and malignant breast tissues [18]. Accordingly, the scientific issue has evolved from “are viruses present in breast cancer” to “are viruses in breast cancer oncogenic or harmless passengers?”

We shall now consider the key candidate viruses and their potential causal role in breast cancer.

## 4. Human Papilloma Virus and Breast Cancer

High risk HPV has been formally recognized as being causal in virtually all cervical cancers. In recent years, evidence has emerged which indicates that HPVs may also have a role in breast cancer. HPV high risk types 16, 18 and 33 in have been identified in breast cancers from widely different populations [19]. We have identified 20 studies which sought to identify HPV in breast cancer that had occurred in a range of populations including Australia, Italy, Norway, China, Japan, USA, Austria, Brazil, Taiwan, Turkey, Greece, Korea, Mexico, Hungary and Syria. The prevalence of HPV positive breast cancer in these studies varied from 4.4% in Mexican to 86.2% in US women. In a Turkish study and our recent study, high risk HPV’s were significantly more prevalent in breast cancer than normal tissues [19,37]. HPV was not identified in normal breast tissue adjacent to breast cancer in the other studies. HPV of the same type have been identified in both breast and cervical cancers that have occurred in the same women [38,39].

In contrast to cervical cancer, HPV is difficult to detect in breast cancer specimens. It is likely that these difficulties are due to very low levels of HPV DNA sequences in breast cancer and the lowered integrity of the DNA in fixed samples which may account for the lack of detection of HPV in several studies [40]. It is also likely that the variations in prevalence of HPV associated breast cancers are due to differences in laboratory methods. In addition the main detection technique–polymerase chain reaction (PCR), is subject to both false positive and negative outcomes mainly because of problems with contamination. Standard, ie liquid PCR methods of detection, were used in all of the 20 studies. Because of the problem of contamination with standard PCR methods, we also used *in situ* PCR in our recent study [36]. *In situ* PCR is also subject to false positive and negative outcomes but is much less liable to contamination than standard PCR.

**Figure 1:**
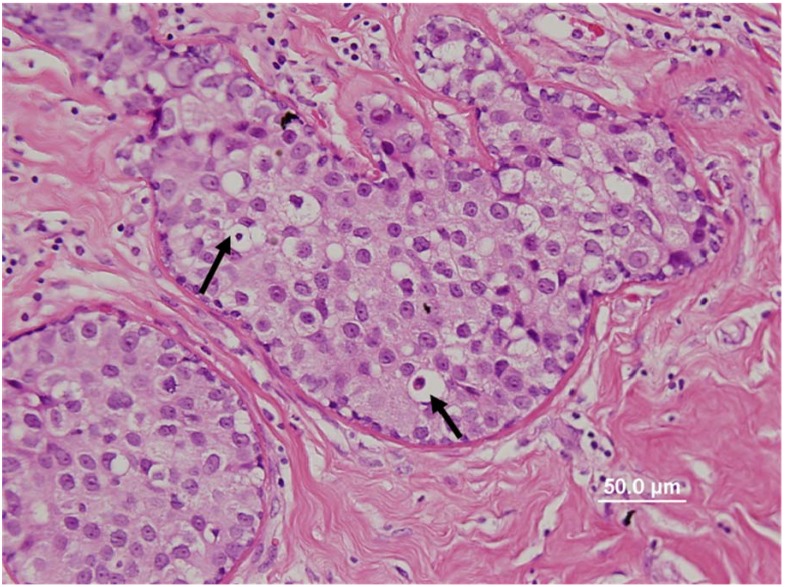
Human papilloma virus associated koilocytes (see arrows for selected koilocytes) in a ductal carcinoma *in situ* breast cancer specimen [18].

In this recent study we have unambiguously demonstrated the presence of high risk HPV-18 in breast cancer cell lines and in the nuclei of breast tumor cells [19]. In addition, we showed the presence of HPV in breast cancer associates with the detectible expression of the HPV E6 oncogenic protein and the p16^INK4A^ protein, which is an indicator of E7-inactivation of the p110^RB^ and that the presence of high risk HPV E6 is significantly associated with low levels of *p53*. Finally, as shown in Figure 1, we identified the presence of HPV associated koilocytes in both breast tumors and normal breast tissues from normal women [18]. In summary, we demonstrated the location of the virus in its’ usual host cell (epithelial cells) and also the expression of its oncoprotein E6, however the interaction role of this oncoprotein with cellular protein has yet to be proven. While not conclusive, these observations suggest HPV is associated with and may even have a causal role in breast cancer.

The oncogenic mechanisms by which HPV induces cervical cancer have been intensively studied. HPV associated cervical cancer can be used as a model for investigating HPV associated breast cancer (Table 4). The biology of HPV in breast cancer is almost identical to that of HPV in cervical cancer. However the much higher viral load in cervical cancer is an important difference. High risk HPV encodes a series of proteins some of which have oncogenic potential. HPV proteins are designated as E1 to E7 (E for early) and L1 and 2 (L for late). E4 disrupts cytoplasmic cytokeratin causing perinuclear cytoplasmic clearing and nuclear enlargement which leads to the appearance of a koilocyte [35]. The influences of HPV E4 usually take place before it is integrated into the human genome. HPV E4 assists in the disruption of cells which release new HPV to the surrounding cells. E6 and E7 oncoproteins stimulate cell cycle progression and disrupt cell cycle regulation by binding to and inhibiting the functions of the proapoptopic and tumor suppressing genes *p53* and *RB-1* which leads to the proliferation of HPV infected basal cells. By binding to the apoptosis inducing (*p53*) protein, E6 also inhibits cell death (apoptosis). HPV E7 induced degradation of p110^RB^ results in a reciprocal over-expression of the cyclin dependent kinase inhibitor p16^INK4A^ [35].

**Table 4:** **HPV-associated cervical and breast cancer**.

	Cervical cancer [41]	Breast cancer [36]
HPV positive	Over 90–95%	0–86%
HPV types	High risk 16/18	High risk 16/18
Koilocyte positive	Approx 40%	Approx 30% [18]
HPV E6 protein inhibits apoptosis (cell death) via p53	HPV E6 inhibits apoptosis via p53	HPV E6 inhibits apoptosis via p53

Steroid hormones, such as estrogens and progesterone, plus HPV infection, enhance cervical epithelial cell transformation [41]. The biological effects of glucocorticoids on HPV-16 mediated human cell carcinogenesis are striking, with a 30-fold increase in growth of immortalized cells following such exposure [42,43]. The influence of hormones on HPV mediated carcinogenesis is relevant to breast cancer, which is estrogen dependent [6].

The immortalization of normal human breast epithelial cells *in vitro* by HPV gene E6 and E7 is compatible with a role for HPV in breast cancer [44].

There is epidemiological evidence which does not support a causal role for HPV in breast cancers. This evidence does not negate the evidence that HPVs are present in breast cancers but does suggest that they may not be causal and are simply parasites in pre-existing cancer tissues. Firstly, the presence of antibodies to HPV 16 in the sera of patients with breast cancer is approximately 10%, which is no different to patients with non-HPV related cancers [45]. The meaning of this observation is far from clear as the presence of HPV 16 associated breast and cervical cancer is well documented and antibodies should be present. Secondly, HPV associated cervical cancer does not increase following migration from Asian countries to the US as does both breast and prostate cancer. This suggests that the increase in breast cancer following such migration may be due to factors other than HPV.

### Methods of Transmission of HPV

The mechanics of HPV transmission in breast cancer is of interest as it is commonly assumed that cell surface to surface contact, mainly during sexual activities, is required. It is possible that there may be initial surface to surface cell contact with HPV virions that are expressed by desquamating cells during sexual intercourse followed by transmission via the blood or lymphatic systems [46,47]. Recently the presence of HPV in circulating white blood cells of 9% of normal male blood donors has been confirmed and suggests a likely mechanism for the spread of HPV to multiple organs [48]. The transmission of HPV infections by oral sexual activities has recently been demonstrated and is also a possible transmission route for HPVs in breast cancer [49]. In a recent Finnish study, a range of high risk for cancer HPVs were identified in all family members including infants an indication that spread of HPV can occur without sexual transmission [50]. In addition, HPV sequences of the same type (type 16), have been identified in breast tumors that occur in women with HPV associated cervical cancer [38,39]. Therefore it is possible that HPVs may be transmitted by hand from the female perineum to the breast, which could occur during sexual activities or even showering or bathing.

## 5. Mouse Mammary Tumor Virus and Breast Cancer

MMTV-like virus has been a major suspect as a cause of some human breast cancers for over 50 years. This is because MMTV is the well established etiologic agent of mammary tumors in field and experimental mice, and MMTV-like gene sequences have been identified in human breast cancers [30,51]. MMTV is transmitted both through mouse milk (exogenous transmission) and through the mouse germ line (endogenous transmission). Despite the substantial evidence that MMTV-like virus may be associated with human breast cancer, development of conclusive evidence has been elusive [2]. The reasons include the difficulty in detecting the low levels of MMTV-like sequences in human breast cancers and concern that the main investigative tool (PCR) may be confounded by false positive and false negative results outcomes [30,52].

We have identified 23 studies conducted in 11 countries which sought to identify MMTV-like virus envelope (*env*) gene sequences in breast cancer specimens. Standard PCR methods were used in each of these studies. MMTV-like gene sequences were identified in breast tumors in 17 of these studies but rarely in normal breast specimens [2]. As previously noted standard PCR methods are subject to contamination and for this reason in our recent study we used *in situ* PCR to supplement standard PCR [19]. This confirmed the finding of the only previous study to use *in situ* PCR to demonstrate that MMTV-like gene sequences were located in the breast cancer cell nuclei [53]. Seventy percent of the complete MMTV-like virus genome, identified in human breast cancer specimens and viral particles from human breast metastases, have been sequenced and shown to display 91% to 99% homology to MMTV from mouse mammary tumors [54]. In a recent study, *env* and LTR sequences with more than 98% homology with those of MMTV have been identified in breast cancers that had occurred in a mother, father and daughter of the same family, living under the same roof [55]. MMTV can infect, integrate and multiply in human breast epithelial cancer cell lines [56,57,58].

We have used the model of MMTV caused mouse mammary tumors to compare the likely life cycle and carcinogenic influences of MMTV in humans [52,59]. This is shown in Table 5. In mice, the main features of MMTV associated tumors include (i) transmission by mother’s milk to newborn pups; (ii) transmission endogenously through the germ line; (iii) MMTV ingestion into the gut and entrance to the lymphatic system through lymphocytes and dendritic cells in the Peyer’s patches; (iv) transmission of MMTV infected lymphocytes to the spleen where they remain dormant for long periods then the infected lymphocytes move to the mammary glands where the MMTV integrates into the DNA of the host mammary epithelial cells. Although integration of MMTV proviral DNA is thought to be essentially random, integration of an MMTV provirus in the vicinity of a number of host oncogenes, particularly near the *Wnt* and *Fgf* family genes, results in inappropriate oncogene expression and clonal outgrowth [59]. When abnormally expressed in mouse mammary tissues, Wnt-1 contributes to hyperplasia and malignant progression.

**Table 5:** Lifecycle of MMTV in mouse and human [51,58].

	Mouse	Human
Transmission	Milk and germ line	Milk/contaminated Food [65,66]
Ingestion	Gut–Peyer's patches	Gut? Peyer’s patches [64]
Immune system	Lymphocytes Superantigens	Superantigens [67]
Tropism	Tropic mammary epithelial cells	Tropic breast epithelial Cells [54]
Latency	Adult mammary tumors	Adult breast cancer
	Sex hormone responsive	Sex hormone responsive [63]

The influence of hormones on MMTV virus is of special interest because of the dependency of human breast cancer on estrogens and other hormones [6]. Estrogens induce mouse mammary tumors in the presence of MMTV but not in its absence [60]. Between 10 to 100 times more virus is produced by corticoid influenced MMTV containing cells, than by controls [61]. In mouse mammary tumors, integration in the vicinity of the *Wnt* (*int*) loci occurs as an early event and the (clonal) growth and the development of the tumor is initially hormone dependent. The tumors become hormone independent and progress presumably because of the accumulation of other genetic insults [62]. This is particularly noteworthy as the prevalence of MMTV-like virus sequences in human gestational breast cancer (cancer occurring during pregnancy or 12 months post partum), is as high as 62% as compared with 30–38% for sporadic breast cancers, which suggests an influence of hormones on MMTV-like viruses also in humans [63].

As suggested above, the lifecycle of MMTV in mice is almost exactly mirrored in humans (Table 5). The main difference is the endogenous transmission of MMTV in mice which does not appear to occur in humans. In addition the oncogenic features of mouse mammary tumors are very similar to those of some breast cancers in humans (Table 6).

It has been shown *in vitro* that, MMTV can contribute to oncogenesis by direct transformation of normal human epithelial cells by expression of signalling proteins [68]. Moreover, evidence has been presented that the MMTV *env* protein participates in mammary epithelial cell transformation *in vivo* using a transgenic mouse model [69]. In a recent study, MMTV was shown to integrate randomly into the genome of human and mouse cultured epithelial cells [58]. However no such integration was identified within 50 kb of either *Wnt* or *Fgf* genes in both the human or mouse genome. This study is in contradiction to the previous evidence and additional oncogenic mechanisms to the influence of MMTV on *Wnt* and *Fgf* may be involved [59].

**Table 6:** MMTV-associated-mouse mammary tumors and human breast cancers [2].

	Mouse mammary tumors	Human breast cancer [19]
MMTV infections	0–100%	0–65% [2]
MMTV positive tumors	25%	15% [19]
Tumor histology	Sheets round cancer cells	Sheets round cancer cells [19]
Tumor molecular structure	LTR/gag/pro/pol/env/LTR-10,000 base pairs	LTR/gag/pro/pol/env/LTR-10,000 base pairs [54]
Oncogenic mechanism	Insertional oncogenesis–near Wnt1 oncogene	Insertional oncogenesis [58]

**Figure 2:**
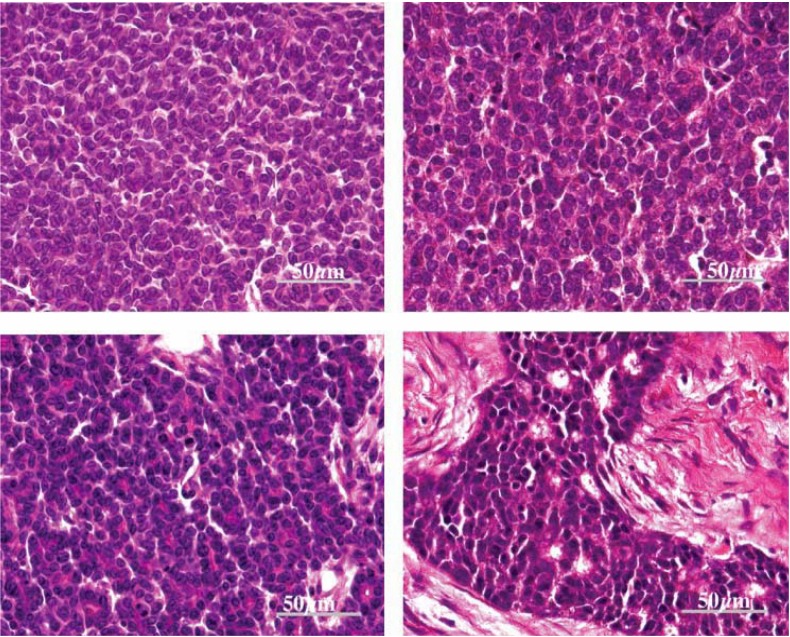
Histological characteristics of MMTV-associated mouse mammary tumors (magnification 600×) compared with human IDC breast cancer specimens. Left (top and bottom): mouse mammary tumor specimens; right (top and bottom): human IDC breast cancer specimens. The histological characteristics of these mouse mammary tumors are virtually identical to the human breast cancer specimens [19,92].

We have recently demonstrated that MMTV gp52 protein and *Wnt1* are highly expressed in some MMTV-like virus positive breast tumors, and that a significant majority of MMTV-like IDC specimens had histological similarities to MMTV positive mouse mammary tumors [19]. This similarity is shown in Figure 2. These findings are consistent with the mouse model of exogenous MMTV associated mouse mammary tumors in wild mice and suggest a similar role in some human breast tumors.

### 5.1. Transmission of MMTV to humans

While there is seemingly consistent evidence that breast fed babies are at no higher risk than non-breast fed babies of developing breast cancer and, at least among epidemiologists, there is a consensus that milk borne viruses are not associated with human breast cancer, spread of MMTV-like virus by human milk is a theoretical possibility [70,71]. This is because it has been realized that virtually all epidemiological studies into breast feeding have been based on questionnaires and mothers respond in varying ways depending on various understandings of the meaning of breast feeding. Current data, based on direct observation, suggests that it is possible that 90% to 100% of newborn babies are “exposed” to colostrum or breast milk despite failure of some mothers to continue breast feeding [71]. Accordingly transmission of MMTV-like virus by human breast milk to new born infants is possible.

MMTV-like particles have been observed in human milk from women with breast cancer and women with a family history of breast cancer [72]. These observations have not been confirmed or replicated and are regarded by many as controversial. However, using PCR, Ford [65] identified MMTV-like *env* gene sequences in some human milk samples from normal Australian women. Recently, Harpreet Johal has identified MMTV *env* sequences in 5% of human milk from normal mothers [66]. Milk borne transmission of retroviruses is complex as has been increasingly demonstrated by the experience of milk borne human immunodeficiency virus (HIV) among African infants. For example HIV milk borne viral transmission is enhanced by consumption of solid foods in addition to milk [73].

As discussed above, continuous zoonotic (animal to human) transmissions of MMTV to humans are a possibility. It has been suggested that the world wide distribution of MMTV-like virus gene sequences in breast cancer in human populations parallels the distribution of the MMTV carrying common house mouse -*Mus domesticus* [16]. Transmission of MMTV by human ingestion of cereal and other food contaminated by mouse faecal material is a speculative possibility [74].

## 6. Epstein-Barr Virus (EBV)

EBV infects almost all of the world’s adult population and establishes a lifelong persistence [75]. EBV has a causal role in Burkitt's and Hodgkin lymphoma and epithelial cell cancers including nasopharyngeal carcinoma [75]. Because EBV infections are ubiquitous in both low and high risk for breast cancer populations, it is unlikely that EBV acts alone to cause breast cancer. On the other hand, as indicated previously, the oncogenic influences of EBV infections contracted during teenage or young adult life in high risk for breast cancer Western countries, appear to be greater than EBV infections that occur during infancy or early childhood [15]. EBV probably collaborates with other factors such as malaria in the case of Burkitt lymphoma and herbal snuff and salted fish in genetically susceptible populations, such as Southern Chinese, in the case of naso-pharyngeal carcinoma. HPVs have been identified in naso-pharyngeal and other head and neck cancers and may also be co-factors with EBV in naso-pharyngeal carcinomas [76,77,78].

The most specific evidence for an association between EBV and breast cancer is the identification of EBV genes within breast cancers. We have identified 27 published studies concerning EBV in breast cancer. Twenty two of the studies were primarily based on polymerase chain reaction (PCR) analyses; EBV sequences were identified in 18 of these studies. Five of the studies were primarily based on immunohistochemistry (IHC) and/or *in situ* hybridization techniques (ISH); EBV was not identified in any of these studies. Only three studies used normal breast tissues from normal women as controls, EBV was not identified in normal breast tissues in two of these studies, in the other study EBV was identified in 23% of cancer and 35% of normal specimens [79]. Evaluation of EBV in breast cancer is difficult because of the extremely low EBV viral loads and the latency of EBV with minimal protein expression [80,81]. Many of the studies of EBVs in breast cancer have not been conclusive, because whole tumor studies based on standard PCR techniques cannot distinguish between cancer cells and infiltrating lymphocytes. This problem has not been completely overcome following the realization that use of the antibody EBNA to identify the cellular location of EBV by Bonnet *et al.* was misleading due to cross reacting proteins [81,82]. However, despite these difficulties, it has become increasingly accepted by workers in this field that EBV can be identified in breast cancer specimens by specific PCR techniques.

EBV has been identified in human milk and transfection of EBV DNA stimulates the growth of human milk cells [83,84]. Normal breast epithelial cells can be infected by direct contact with EBV containing lymphatic cultured cell lines [85].

The oncogenic mechanisms for EBVs appear to differ with respect to lymphatic cells (leading to lymphomas) and epithelial cells (leading to naso-pharyngeal and other carcinomas). It is of special interest that one oncogenic mechanism for EBV has recently been identified by use of gene knockout techniques [86]. Schneider *et al*. demonstrated that the TRADD (TNF-receptor 1-associated death domain protein) gene is an essential factor for the function of the EBV virus encoded latent membrane protein 1(LMP1) which causes uncontrolled proliferation of EBV infected cells and ultimately cancer [86]. The EBV BARF1 gene causes epithelial cell immortalization in experimental monkeys and is expressed in EBV related human gastric and nasopharyngeal carcinomas, which suggests an oncogenic role for this gene [87]. EBV is known to promote epithelial cell growth [87,88].

In Hodgkin’s lymphoma and infectious mononucleosis, EBV associated Reed Sternberg (RS) cells may be present. These are abnormally shaped cells which may contain multiple nuclei and which originate from B-lymphocytes [89]. RS cells have been identified histologically in invasive breast tumors [90].

There is no exact model with which to compare the biology of EBV associated breast tumors. We have used EBV associated Hodgkin's lymphoma to demonstrate some of the common features with EBV associated breast cancer in Table 7. It will be noted that while RS cells may be present in both Hodgkin's lymphoma and breast cancer, EBV may infect both epithelial and lymphatic cells in breast cancer but only lymphocytes in lymphomas. A crucial unknown is that EBV is such a common infection and which is known to increase the risk of Hodgkin's lymphoma, but the development of lymphomas and to a lesser extent breast cancer, are comparatively rare events.

**Table 7:** Epstein-Barr virus comparison Hodgkin's lymphoma and breast cancer [2].

	Hodgkin's lymphoma	Breast cancer
Age of most EBV infections	Teenage–young adult Western	Teenage–young adult Western females [15]
Malignant cell type	B lymphocytes	Putative lymphocytes and epithelial cells [81]
EBV histology	Reed/Sternberg cells	Reed/Sternberg cells [90,91]

## 7. Bovine Leukemia Virus and Breast Cancer

Work in this field has been initiated by just one scientist- Gertrude Buehring of the University of California at Berkeley. Since the main source of milk for humans is cows, she reasoned that a bovine virus might be a likely candidate for a milk-transmitted agent of human breast cancer. Cattle are commonly infected with bovine leukemia virus (BLV), a cancer causing virus which can be transmitted from cow to calf via the milk. Most infected cattle are healthy and are not removed from the herd. Consumption of non-pasteurized dairy products or undercooked beef could conceivably allow transmission of infectious virus to humans. BLV can infect other species including sheep and goats naturally, and several species experimentally, including non-human primates. BLV can also infect the cells of many species cultured in flasks, including cells from humans and other primates. BLV infects the mammary epithelial cells of cows naturally and in culture [32]. This indicates that this "leukemia" virus can target more than just blood cells.

Buehring has demonstrated that 39% of humans in a San Francisco Bay Area population have antibodies to BLV in their blood, which is an indication of exposure to BLV [33]. In a study of 213 subjects using *in situ* PCR, BLV-related DNA was detected significantly more frequently (59%) in breast tissues from women with a diagnosis of breast cancer than in breast tissues from women with no history of breast cancer (29%) [34]. Caution is required when considering a possible role of BLV, as it has been observed that antibodies to retrovirus proteins such as BLV are not specific for breast cancer.

### Multiple viruses in human breast cancer

Cytomegalovirus (CMV) and Simian virus 40 (SV40) have also been identified in human breast cancers [2]. This is not surprising as milk production is dependent on hormones which in turn promote the replication of many viruses. The challenge is to determine if any of these many viruses are oncogenic in humans.

## 8. Overall Interpretation of the Evidence

The evidence that high risk HPVs and MMTV-like viruses are present in human breast tumors is in our view, conclusive. The evidence for the presence of EBV in human breast cancer is substantial but not conclusive. More research is required to determine whether BLV is present in human breast cancer. Whether these viruses are causal rather than passengers or invaders of existing malignant tissues, has not been established.

When considered in the context of the molecular based evidence, the presence of HPV associated precancerous koilocytes in normal and breast tumor tissues is very suggestive of a causal role. Similarly, the lifecycle and oncogenic characteristics of MMTV-like virus in humans is also suggestive of a causal role. The evidence for a causal role of EBV is less substantial but the identification of EBV Reed Sternberg cells in association with the molecular based evidence is suggestive.

If conclusive evidence for a causal role for these viruses can be developed, there will be a rational explanation of the heterogeneity of human breast cancer and above all a practical possibility of effective preventive and treatment approaches.
